# Editorial: Reproductive Events in Women With Mood Disorders: Advances in Knowledge and Management

**DOI:** 10.3389/fpsyt.2021.767983

**Published:** 2021-11-30

**Authors:** Gianluca Rosso, Prabha S. Chandra

**Affiliations:** ^1^Department of Neurosciences Rita Levi Montalcini, University of Turin, Turin, Italy; ^2^Psychiatric Unit, San Luigi Gonzaga University Hospital, Turin, Italy; ^3^Departement of Psychiatry, National Institute of Mental Health and Neuroscience (NIMHANS), Bangalore, India

**Keywords:** mood disorders, women, peripartum period, sexual hormones, reproductive cycle events

A growing body of evidence suggests a relationship between the course of affective disorders in women and reproductive cycle events such as menarche, pregnancy/delivery and menopausal transition (1, 2). Particularly, the peripartum period is considered at high risk for mood instability in women with a history of major depressive disorder (MDD) and bipolar disorder (BD) especially (3, 4). Several factors related to this crucial period, including biological, cognitive and psychosocial aspects (especially, maternal stressful events during the pregnancy and the postpartum period), contribute to the women's vulnerability to mood episodes and the resulting obstetrical and neonatal complications (5, 6). On the one hand, fluctuating ovarian hormones may affect the perception of emotional information and thus the mood and the cognitive responses to such information. In addition to the impact of estrogens on emotional processing, reproductive hormones may also modulate other cognitive processes which are altered in mood disorders, such as attention and processing speed (5). On the other hand, the variations of mood may be affected by specific neurophysiological abnormalities in sub-regions of the prefrontal cortex of BD patients, which could make women more vulnerable to hormonal changes and to peripartum relapses due to the role of such regions in the neurocognitive dysregulation of emotion (7).

The aim of this Research Topic was to collect original contributions by research groups from different countries, in order to give new insights on the interactions between female reproductive events and mood disorders. Eight articles were gathered: one observational study and one case report concerning menstrual cycle; four observational studies, one literature review, and one clinical trial concerning the peripartum period.

While the impact of menstrual cycle and menopause on the course of mood disorders have been extensively studied, findings concerning correlation between age at menarche and mental illnesses are scarce. In their cross-sectional study involving a large sample of BD patients, Rosso et al. assessed the relationship between timing of menarche and course of disease, including peripartum recurrences and medical comorbidities. Interestingly, women with late menarche had a lower risk of peripartum affective episodes and women with early menarche had higher rates of metabolic syndrome. These findings suggest a possible link between fluctuations of sex hormones during menarche and mental and physical health of women, which deserves to be deepened in further studies.

On the other hand, a personalized risk-benefit evaluation of treatments interfering with hormonal regulation of female reproduction, such as contraceptives, should be conducted, taking into account their influence on mood state. To this end, Zeiss et al. presented the case of a woman who developed anxiety and depressive symptoms after replacement of a common contraceptive intrauterine device, highlighting the necessity to discuss with women the potential psychiatric side effects of hormonal contraception.

Several studies reported on the risk of affective recurrences during the peripartum period, especially in the postpartum period (8). Phua et al. examined how maternal mental health problems vary during and after pregnancy. Depressive and anxiety symptoms appeared densely connected in both pre and postpartum period, but even more strongly after delivery, due to their mutual reinforcement over the course of pregnancy. Moreover, the authors found qualitative differences in core anxious-depressive symptomatology across the peripartum period: sense of worthlessness or uselessness were predominant during pregnancy, while feelings of “being overwhelmed” or “punished for being a bad mother” became central after delivery. Anxiety was found to be a bridging symptom preceding the development of postpartum depression (PPD) and may result a key target for reducing the risk of PPD and improving its prognosis.

Over the years, it was observed that psychological health of mothers may have consequences for fetal/neonatal neurobehavioral development and maternal newborn care. Boekhorst et al. found women with BD to be more vulnerable to mother-to-infant bonding impairment in the first year postpartum, regardless of the occurrence of postpartum relapse. According to the authors, these effects might be mediated by the nature of their disorder itself (related to feelings of inadequacy or insecurity and negative cognitions perceived by pregnant women with BD).

The increased risk of onset and relapse of mood disorders in women of childbearing age is well-known, nevertheless a more accurate identification of predictive factors to early recognize perinatal episodes is awaited. Khan et al. tried to indentify different psychosocial and personal variables related to the risk of prenatal depression. Prior pregnancies, living in a joint family, feelings of dissatisfaction, stressful life events, or psychological abuse by partners were associated with higher rates of prenatal depression. This may be related to the fact that stress can influence the expression and severity of mood disorders in the peripartum period. Focusing on maternal psychological distress, the cross-sectional study by Shiva et al. found that, in a sample of Indian women at their first pregnancy, complications during delivery and traumatic childbirth were associated with post-traumatic stress disorder (PTSD) and depression in the postpartum period. Moreover, the review by Yu et al. focused on triggering factors related to PPD, such as reproductive or stress hormones and inflammation, with the aim of identifying potential biological markers for PPD. Progesterone levels, which progressively increase throughout pregnancy and rapidly drop after delivery, were confirmed to be correlated to PPD; interestingly, the authors showed that a low prenatal allopregnanolone (a metabolite of progesterone which varies proportionally with progesterone levels) predicts PPD. The authors concluded that we need more research to clarify the existence of associations between the other risk factors analyzed and PPD. Given the lack of biological and clinical predictors, these findings may help to early recognize and appropriately treat perinatal recurrences.

The decision to treat mood disorders during the peripartum period should be carefully considered, balancing the risk of prenatal and neonatal exposure to medication vs. the potentially deleterious effects of untreated affective disorders on the fetus/child (9, 10). Accumulating evidence shows that mood stabilizers (particularly lithium) may prevent affective recurrences without significant increase of teratogenic risk and with few side effects to both mothers and babies (11, 12). Similarly, selective serotonin reuptake inhibitors may help minimize adverse maternal and neonatal outcomes and are generally well-tolerated (13, 14). Brouwer et al. investigated the effect of tapering antidepressants in pregnant woman who received preventive cognitive therapy (PCT), as compared to those continuing antidepressants, and found no significant differences in affective fluctuations nor prenatal relapse rates, as women receiving PCT were able to regulate emotions lowering the risk of depressive recurrences. The authors also explored whether mood fluctuations in early pregnancy could affect neonatal weight and, in contrast to previous findings, didn't find significant correlations.

In conclusion, the high-quality contributions to this Research Topic covered several original aspects of the impact of reproductive events on the course of affective disorders, including the role of stress and hormones also as potential predictors of mood episodes. It's time to introduce screening programmes for women's mental health in the context of preconception counseling and in obstetrics and gynecology ward, in order to ensure early detection of mood disturbances and guarantee continuity of care in reproductive age and in the peripartum period.

## Author Contributions

GR and PC wrote the editorial and approved the submitted version. Both authors contributed to the article and approved the submitted version.

## Conflict of Interest

GR has been a speaker and/or consultant from Angelini, Janssen, Lundbeck, and Otsuka. The remaining author declares that the research was conducted in the absence of any commercial or financial relationships that could be construed as a potential conflict of interest.

## Publisher's Note

All claims expressed in this article are solely those of the authors and do not necessarily represent those of their affiliated organizations, or those of the publisher, the editors and the reviewers. Any product that may be evaluated in this article, or claim that may be made by its manufacturer, is not guaranteed or endorsed by the publisher.
